# Mechanisms of Human Innate Immune Evasion by *Toxoplasma gondii*

**DOI:** 10.3389/fcimb.2019.00103

**Published:** 2019-04-16

**Authors:** Tatiane S. Lima, Melissa B. Lodoen

**Affiliations:** Department of Molecular Biology and Biochemistry and the Institute for Immunology, University of California, Irvine, Irvine, CA, United States

**Keywords:** *Toxoplasma gondii*, immune evasion, innate immunity, IFN-γ, pro-inflammatory cytokines, apoptosis

## Abstract

*Toxoplasma gondii* is an intracellular protozoan parasite of global importance that can remarkably infect, survive, and replicate in nearly all mammalian cells. Notably, 110 years after its discovery, Toxoplasmosis is still a neglected parasitic infection. Although most human infections with *T. gondii* are mild or asymptomatic, *T. gondii* infection can result in life-threatening disease in immunocompromised individuals and in the developing fetus due to congenital infection, underscoring the role of the host immune system in controlling the parasite. Recent evidence indicates that *T. gondii* elicits a robust innate immune response during infection. Interestingly, however, *T. gondii* has evolved strategies to successfully bypass or manipulate the immune system and establish a life-long infection in infected hosts. In particular, *T. gondii* manipulates host immunity through the control of host gene transcription and dysregulation of signaling pathways that result in modulation of cell adhesion and migration, secretion of immunoregulatory cytokines, production of microbicidal molecules, and apoptosis. Many of these host-pathogen interactions are governed by parasite effector proteins secreted from the apical secretory organelles, including the rhoptries and dense granules. Here, we review recent findings on mechanisms by which *T. gondii* evades host innate immunity, with a focus on parasite evasion of the human innate immune system.

## Host Innate Immunity to *T. gondii* Infection

*Toxoplasma gondii* is an obligate intracellular protozoan parasite that infects an estimated one-third of the global human population. Although most infections are asymptomatic, *T. gondii* can cause life-threatening infections in immunocompromised individuals and the developing fetus (Montoya and Liesenfeld, 2004). During infection, *T. gondii* disseminates via the circulatory system and establishes chronic infection in several organs, including the heart and brain (Harker et al., 2015).

Although both humans and rodents are hosts for *T. gondii*, there are key differences in the innate immune responses between these species. In the mouse, innate immunity is mediated by TLR11 and TLR12 recognition of *T. gondii* profilin, which is the dominant mechanism driving IL-12 production (Yarovinsky et al., 2005; Koblansky et al., 2013). Notably, TLR11 is non-functional in humans, and TLR12 does not exist in the human genome, indicating alternative mechanisms of parasite sensing in human cells. Although these mechanisms have not been completely defined, it is known that IL-12 is produced by human neutrophils and monocytes in response to *T. gondii* (Bliss et al., 1999; Aldebert et al., 2007). In human monocytes, unlike in mouse macrophages (Robben et al., 2004), phagocytosis of the parasite drives this IL-12 response (Tosh et al., 2016). IL-12 induces the production of IFN-γ, a key mediator of immunity in humans and mice (Suzuki et al., 1988; Ceravolo et al., 1999) that initiates protective type 1 immunity (Gazzinelli et al., 1994; Däaubener et al., 1995).

In addition to activating T cell-mediated immunity, IFN-γ functions in a cell-autonomous manner to control intracellular parasites. IFN-γ increases tryptophan degradation in human fibroblasts, inhibiting parasite replication (Pfefferkorn, 1984). More recently, IFN-γ inducible proteins were found on the parasitophorous vacuole membrane (PVM). Interestingly, these immunity-related GTPases (IRGs) play an important role in cell-intrinsic antimicrobial defense in the mouse (Zhao et al., 2009), but this locus is considerably smaller in humans and does not appear to be involved in immune defense. A parallel IFN-γ-dependent mechanism of resistance in humans and mice consists of the guanylate binding proteins (GBPs), which are recruited to the parasitophorous vacuole membrane and cause vacuolar membrane disruption and parasite clearance (Yamamoto et al., 2012; Degrandi et al., 2013; Selleck et al., 2013). Human GBP1 restricts replication of type II *T. gondii* in epithelial cells without targeting the parasitophorous vacuole (Johnston et al., 2016), suggesting that GBPs can participate in host defense without causing classical vacuolar membrane disruption. In human cells, ubiquitination at the parasite vacuole has also emerged as a key mechanism of parasite control, leading to non-canonical autophagy and parasite growth stunting in HeLa cells (Selleck et al., 2015) or endolysosomal fusion and parasite clearance in umbilical vein endothelial cells (Clough et al., 2016).

As an obligate intracellular parasite, *T. gondii* has evolved strategies to successfully manipulate the host immune system to establish a productive infection and maintain an optimal replicative niche. Here we review recently described strategies by which *T. gondii* specifically evades human innate immunity, with brief mention of related studies in the mouse.

### Modulation of Host Signaling Pathways

**Figure 1:**
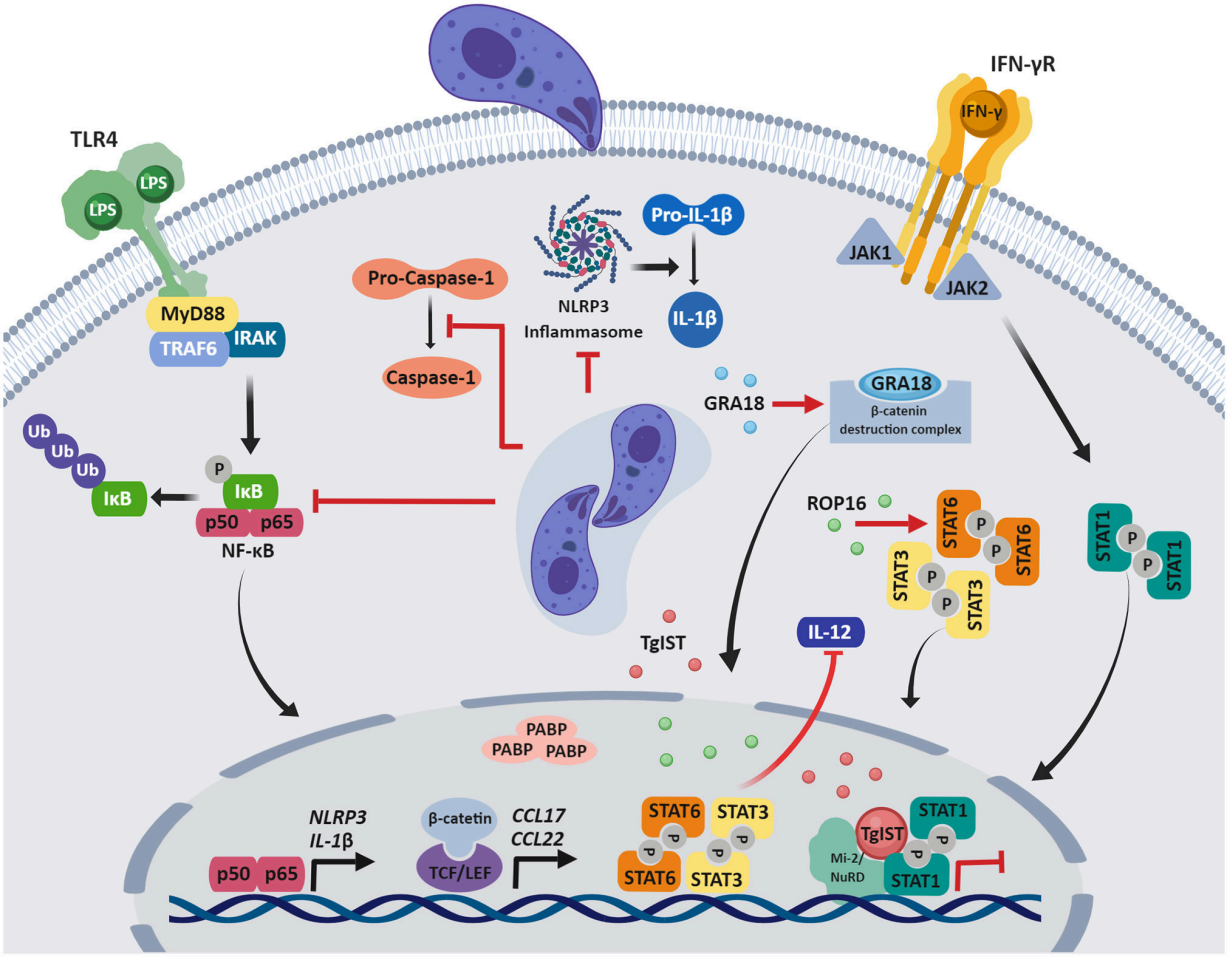
Modulation of host immune signaling by *T. gondii*. After invasion of the host cell, *T gondii* manipulation of host signaling pathways and gene expression impair innate immune responses. Parasite effector proteins that govern many of these host-pathogen interactions are secreted from the apical secretory organelles and are found in the host cytosol, associate with the PVM, or translocate to the host nucleus. Although a variety of mechanisms of immune evasion are shown, it should be noted that the function of specific effector proteins may depend on the *T gondii* strain and human cell type that is infected, as described in the review. Figures were created using BioRender.

The manipulation of signaling pathways leading to cytokine production is an effective strategy to impair immune responses that compromise pathogen survival. Although *T. gondii* resides within a vacuole in infected cells, effector proteins released through the parasite's specialized secretory organelles, the rhoptries or dense granules, are instrumental in manipulating host cell signaling and transcriptional responses (Figure 1).

The three dominant clonal lineages of *T. gondii* (types I, II, III) notably differ in their effects on host cells. Type I and III, but not type II strains activate the signal transducer and activator of transcription 3 and 6 (STAT3 and STAT6) in human and mouse cells, thereby down-regulating IL-12 (Saeij et al., 2007). Similarly, in mouse macrophages, constitutive activation of STAT3 by type I strains prevents LPS-induced IL-12p40 production (Butcher et al., 2005). The rhoptry kinase ROP16 is responsible for these effects, by phosphorylating and activating STAT3 and STAT6 in human and mouse cells (Saeij et al., 2007; Yamamoto et al., 2009; Ong et al., 2010).

*T. gondii* infection induces a robust IFN-γ-driven immune response that is critical to resolve acute infection and control chronic infection (Suzuki et al., 1988, 1989). IFN-γ stimulation induces a vast transcriptional program (Platanias, 2005), and genome-wide microarray analysis in human foreskin fibroblasts (HFFs) revealed that *T. gondii* infection blocks up-regulation of all 127 genes that were induced by IFN-γ treatment in this study (Kim et al., 2007). Subsequent research determined that type I, II and III strains inhibit STAT1 transcriptional activity through mechanisms independent of ROP16 or GRA15, a dense granule protein that activates sustained NF-κB signaling (Rosowski et al., 2011; Rosowski and Saeij, 2012). IFN-γ stimulation initiates JAK/STAT signaling, whereby STAT1 homodimers translocate to the nucleus and bind to gamma-activated sequences (GAS) in DNA to activate transcription (Sadzak et al., 2008). Notably, *T. gondii* inhibits the expression of IFN-γ responsive genes by preventing the dissociation of STAT1 from DNA, hampering its recycling and further cycles of STAT1-mediated transcription (Rosowski et al., 2014). Recent studies identified a parasite factor conserved among *T. gondii* strains that is required for blocking transcription of IFN-stimulated genes in HFFs: *T. gondii* inhibitor of STAT1-dependent transcription (TgIST) is a dense granule protein that binds to activated STAT1 dimers in the nucleus of IFN-γ-treated cells and also to the chromatin-modifying Mi2/NuRD complex, resulting in altered chromatin and blockade of IFN-γ-dependent transcription (Gay et al., 2016; Olias et al., 2016). Ectopic expression of TgIST in human cells demonstrated that it is sufficient to repress STAT1-dependent promoter activity (Gay et al., 2016). Moreover, in IFN-γ-treated mouse macrophages, TgIST blocks IRG-mediated clearance of type II *T. gondii* (Gay et al., 2016).

Another major signaling cascade dysregulated by *T. gondii* is the NF-κB pathway, which leads to the production of pro-inflammatory cytokines involved in host immunity. In infected HFFs, type I *T. gondii* limits NF-κB activation by reducing p65/RelA phosphorylation and translocation to the nucleus (Shapira et al., 2005). Type I *T. gondii* also inhibits LPS-induced IL-1β production in primary human neutrophils, and this effect is associated with inhibition of NF-κB signaling. In *T. gondii*-infected neutrophils, IκBα degradation and p65/RelA phosphorylation are reduced, as are transcripts for *IL-1*β and the inflammasome sensor *NLRP3*. *T. gondii* also inhibits caspase-1 cleavage and activation in infected neutrophils (Lima et al., 2018), but not in infected human monocytes (Gov et al., 2013, 2017), representing different human cell type-specific mechanisms of IL-1β regulation.

Recently, GRA18 was identified as a dense granule protein that reprograms inflammatory responses. GRA18 forms complexes with regulatory elements of the β-catenin destruction complex, which includes β-catenin, GSK3α/β, and the PP2A-B56 holoenzyme, promoting stabilization and nuclear translocation of β-catenin, and inducing β-catenin-dependent gene expression (He et al., 2018). β-catenin is the main effector of the Wnt pathway, functioning as a coactivator of the T-cell factor/lymphoid enhancer factor (TCF/LEF) transcription factors (Cadigan and Waterman, 2012). In murine macrophages, GRA18 induces β-catenin-dependent genes associated with anti-inflammatory responses, including CCL17 and CCL22 (He et al., 2018), which may counterbalance type I inflammatory responses.

### Inhibition of Apoptosis

**Figure 2:**
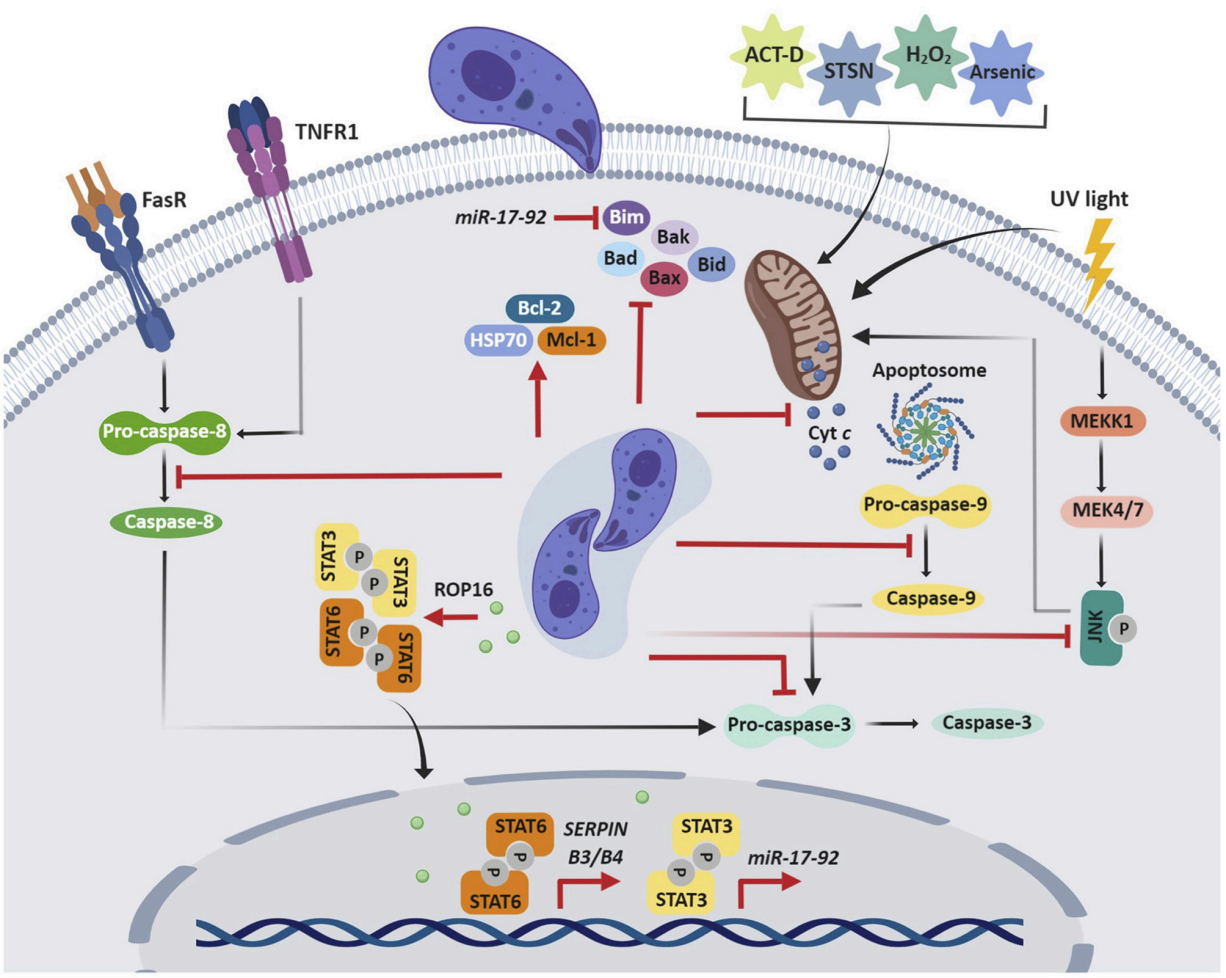
*T. gondii* inhibition of host cell apoptosis. *T gondii* impairs both cell-intrinsic (mitochondrial) and -extrinsic (death receptor-mediated) pathways of apoptosis, which allows the parasite to maintain its replicative niche. *T gondii* can interfere with the initiation, activation, or signaling of the apoptotic cascade, which may result from an indirect mechanism or the direct effect of secreted parasite effector proteins. ACT-D, actinomycin D; STSN, staurosporine; Cyt c, cytochrome *c*.

Although cell death caused by infection can be detrimental to the host, apoptosis is also an important means of eliminating intracellular pathogens (Williams, 1994). Perhaps unsurprisingly, viruses, bacteria, and parasites have evolved strategies to inhibit this programmed cell death (Friedrich et al., 2017). Indeed, *T. gondii* can arrest both cell-intrinsic (mitochondrial) and extrinsic (death receptor-mediated) pathways of apoptosis (Figure 2) in the cells it has invaded. This may help the parasite to preserve its intracellular niche, replicate, and avoid clearance by humoral immunity.

The initial observations that *T. gondii* inhibits host cell apoptosis were in mouse cell lines (Nash et al., 1998); however, over the last 20 years, multiple studies have revealed these effects in human cell lines and primary cells. Collectively, these data show that both type I and II *T. gondii* inhibit the extrinsic and intrinsic apoptotic pathways through similar mechanisms. The first study on human cells demonstrated that *T. gondii*-infected HL-60-derived macrophages are protected from actinomycin D-induced apoptosis (Goebel et al., 1999). This effect on the mitochondrial apoptotic pathway is associated with inhibition of cytochrome *c* release, which in turn reduces cleavage of apoptotic caspase-9 and caspase-3. In addition, Mcl-1, an anti-apoptotic factor from the Bcl-2 family is up-regulated by *T. gondii* infection (Goebel et al., 2001). *T. gondii* inhibition of UV-induced apoptosis of infected HeLa cells is also associated with decreased cytochrome *c* release and apoptotic caspase activity (Carmen et al., 2006). This pathway is known to rely on c-Jun NH2-terminal kinase (JNK) signaling (Tournier et al., 2000), and indeed, JNK activity was repressed in *T. gondii*-infected cells. Subsequent studies of staurosporine-treated HeLa cells and human Jurkat T cells provided evidence for how *T. gondii* impairs cytochrome *c* release. The oligomerization of the Bcl-2 pro-apoptotic proteins Bax and Bak permeabilizes the mitochondrial membrane, allowing the release of apoptogenic proteins, including cytochrome *c* (Jürgensmeier et al., 1998; Annis et al., 2005). Although *T. gondii* infection does not affect Bax or Bak expression, it inhibits conformational changes in these proteins, translocation of Bax from the cytosol to the mitochondria, and oligomerization of Bax, which contributes to decreased cytochrome *c* release (Hippe et al., 2009). Similarly, in arsenic trioxide-treated THP-1 macrophages, *T. gondii* increases expression of Bcl-2 and the anti-apoptotic chaperone heat-shock protein 70 (HSP70), which in turn reduces cytochrome *c* release and caspase-3 activation (Hwang et al., 2010). In staurosporine-treated cells, the mechanism is associated with induction of the serine protease inhibitors B3 and B4 (SERPIN B3/B4) via STAT6 activation (Song et al., 2012). In Jurkat cells, *T. gondii* inhibits apoptosis mediated by granzyme B, a death-inducing serine protease, by inhibiting granzyme B activity (Yamada et al., 2011).

The anti-apoptotic effects of *T. gondii* in diverse cell types appear to converge on inhibition of cytochrome *c* and apoptotic caspases. Interestingly, in a cell-free system with Jurkat cell extracts, the parasite can directly affect cytochrome *c*-induced caspase activation, independent of cytochrome *c* release from host cell mitochondria or upregulation of antiapoptotic molecules (Keller et al., 2006). Notably, parasite lysates mediated this effect, suggesting that a soluble parasite molecule specifically interferes with cytochrome *c*-induced caspase activation (Keller et al., 2006). Binding of cytochrome *c* and of dATP or ATP to the protease activating factor 1 (Apaf-1) allows the formation of a wheel-like heptameric complex, the apoptosome, which in turn activates caspase-9 (Reubold et al., 2009). Interestingly, *T. gondii* inhibits the binding of caspase-9 to Apaf-1, which prevents caspase-9 activity and subsequent caspase-7 and caspase-3 activation (Graumann et al., 2015).

In addition to blocking the intrinsic pathway, *T. gondii* also inhibits the extrinsic pathway of apoptosis. *T. gondii* prevents apoptosis in infected U937 monocytic cells treated with TNF-α and cycloheximide (Goebel et al., 2001). Fas/CD95-induced apoptosis is blocked in the human B cell line SKW6.4 by *T. gondii* interference with the initiator caspase-8, in the absence of a mitochondrial amplification loop (Vutova et al., 2007). Reduced levels of pro-caspase-8 decrease its association with the death-inducing signaling complex (DISC) and impair activation of effector caspases (Vutova et al., 2007). In HeLa cells, in which Fas/CD95-ligation is amplified via the mitochondrial amplification loop, *T. gondii* inhibits cleavage of the pro-apoptotic BH3-only protein Bid, the release of cytochrome *c*, and the activity of the initiator caspase-8 and caspase-9 and the effector caspase-3 and caspase-7 (Hippe et al., 2008).

All of the previously noted human studies characterize the anti-apoptotic effect of *T. gondii* in human cell lines; however, more recently, this effect has been demonstrated in primary human cells. *T. gondii* infection of human peripheral blood mononuclear cell (PBMCs)-derived macrophages blocks staurosporine-induced apoptosis via increased expression of the *miR-17-92* gene cluster (Cai et al., 2014). The promoter of this cluster contains two putative STAT3 binding sites, and *T. gondii* TgCtwh3 with atypical genotype China 1, activates STAT3, similar to type I *T. gondii*. STAT3 activation leads to increased *miR-17-92* expression and decreased expression of Bim, a BH3-only member of the Bcl-2 family that contributes to pore formation in the mitochondrial membrane and cytochrome *c* release (O'Connor et al., 1998; Cai et al., 2014). The miRNA *miR-20a* is a member of the miR-17-92 gene cluster and its expression is up-regulated in human macrophages infected with type I *T. gondii*. Inhibition of this miRNA reverses the effect, resulting in apoptosis of human macrophages (Rezaei et al., 2018).

Glycosylphosphatidylinositols (GPIs) are glycolipids that link proteins to eukaryotic cell membranes. GPI anchors are abundantly expressed on many protozoan parasite surfaces, including *T. gondii* (Lekutis et al., 2001). Since exposing macrophages to *Trypanosoma cruzi* GPIs enhances expression of the anti-apoptotic A1 and Bcl-2-like genes (Ropert et al., 2002), a similar mechanism for *T. gondii* GPIs was investigated; however, highly purified *T. gondii* GPIs do not affect apoptosis of HL-60, Jurkat, or SKW6.4 cells (Debierre-Grockiego et al., 2007). Despite the many studies describing the anti-apoptotic effect of *T. gondii* in human cells, the parasite factor(s) that trigger this response remain unknown.

### Evading Intracellular Death

In phagocytes, such as neutrophils and macrophages, ROS production is an important antimicrobial response for the elimination of pathogens. Interestingly, however, ROS is not induced in *T. gondii*-infected human macrophages (Wilson et al., 1980), potentially due to an immune evasion mechanism, as noted below. Non-phagocytic cells also generate low levels of ROS (Bedard and Krause, 2007), and in ARPE-19 cells, *T. gondii* targets the main NADPH oxidase by reducing Nox4 at the transcript and protein levels, resulting in decreased intracellular ROS. The effect on Nox4 expression was associated with activation of PI3K/AKT signaling in infected cells (Zhou et al., 2013). Proliferation of type I *T. gondii* in murine inflammatory macrophages was also associated with decreased ROS production (Shrestha et al., 2006). Recent studies in mouse macrophages showed that clearance of type III, but not type I, *T. gondii* relies on NADPH activity, increased ROS production, and induction of GBP5, suggesting that virulent strains may block ROS production, enabling parasite survival (Matta et al., 2018).

Microbicidal enzymes also contribute to destroying intracellular and extracellular pathogens. Neutrophil granule enzymes are secreted into the phagolysosome and released during NETosis. A Kazal family serine protease inhibitor, *T. gondii* protease inhibitor 1 (TgPI-1), is secreted by the dense granules and inhibits neutrophil elastase activity (Morris et al., 2002). It is known that both tachyzoites and bradyzoites are resistant to physiological concentrations of trypsin (Sharma and Dubey, 1981), which the parasite encounters in the intestine. TgPI-1 also inhibits trypsin and chymotrypsin, two proteolytic enzymes of the small intestine (Pszenny et al., 2000; Morris et al., 2002). Together, these data suggest a role for TgPI-1 in evading neutrophils and in protecting the parasite in the gut.

### Establishment of a Replicative Niche

*T. gondii* also affects the cell cycle in infected human cells. In HFFs, the parasite induces progression from G0/G1 to S phase and an arrest toward G2/M (Molestina et al., 2008). This response is associated with sustained activation of extracellular signal-regulated kinase (ERK) signaling, which may act as a positive feedback to maintain HFFs in S phase (Molestina et al., 2008). Similar G2 arrest was observed in the human BeWo trophoblast cell line and in primary normal dermal human fibroblasts (NHDFs). *T. gondii* infection induces expression of the E3 ubiquitin-protein ligase UHRF1 and down-regulates the cyclin B1, which may cause the cell cycle arrest (Brunet et al., 2008). GRA16 is a dense granule protein that is exported from the PV into the cytoplasm and accumulates in the host nucleus. This protein binds to two host enzymes, the deubiquitinase HAUSP and the PP2A phosphatase, which regulate p53 and cell cycle, suggesting that GRA16 controls host cell arrest in G2/M phase (Bougdour et al., 2013). Modulation of the host cell cycle may influence how *T. gondii* controls its own replication and suggests a preference for proliferation in G2/M phase.

The first microarrays performed on *T. gondii*-infected cells revealed up-regulation of host genes involved in nutrient scavenging and metabolism, which the parasite requires for replication (Blader et al., 2001). Interestingly, the hypoxia-inducible factor-1 alpha (HIF-1α) transcription factor, which is required for parasite replication, is stabilized and activated in *T. gondii*-infected HFFs (Spear et al., 2006). HIF-1 stabilization occurs because PHD2, a prolyl hydroxylase that targets HIF-1 for proteasomal degradation, is down-regulated during infection via activin-like receptor kinase signaling (Wiley et al., 2010).

Poly-adenosine-binding proteins (PABPs) are RNA binding proteins that bind to polyadenylated RNA and are involved in metabolic pathways of the mRNA, and their sub-cellular distribution changes in response to cellular stress (Gray et al., 2015). Nuclear granulation of PABPs is induced in *T. gondii*-infected HFFs (Fischer et al., 2018), which may enable the parasite to influence the host cell transcriptome. Quantitative proteomic analysis of HFFs also indicates that *T. gondii* globally reprograms key metabolic pathways in the host cell, including glycolysis, lipid, and sterol metabolism, mitosis, apoptosis, and structural-protein expression (Nelson et al., 2008). Together, these processes may facilitate *T. gondii* establishment of its replicative niche.

## Concluding remarks

In the last decade, significant progress has been made in characterizing mechanisms of immune evasion by *T. gondii*. Rodents are a natural host for *T. gondii* and a relevant model for studying many aspects of parasite immunity. However, the extension of these studies to human cells, which differ in key innate immune pathways, will be critical for understanding determinants of human disease. Future work on the contribution of parasite effector proteins to host-pathogens interactions in both hematopoietic and non-hematopoietic human cells will be of particular interest, as will studies investigating the synergistic effects of these proteins or their role in establishing chronic infection, potentially by altering pathways in brain cells (Schlüter et al., 2001; Xiao et al., 2011; Mammari et al., 2014). Ultimately, elucidation of the molecular mechanisms governing human immune evasion by *T. gondii* may provide new insights into potential therapeutic targets that contribute to reduced disease and improved outcomes for human health.

## Author Contributions

TSL wrote the first draft of the manuscript. TSL and MBL edited and revised the manuscript. Both authors read and approved the submitted version.

### Conflict of Interest Statement

The authors declare that the research was conducted in the absence of any commercial or financial relationships that could be construed as a potential conflict of interest.
